# Self-objectification and career aspirations among young Chinese women: the roles of self-esteem and career decision-making self-efficacy

**DOI:** 10.3389/fpsyg.2023.1193008

**Published:** 2023-06-30

**Authors:** Qingqing Sun

**Affiliations:** Quality Education Center, Henan University of Economic and Law, Zhengzhou, China

**Keywords:** self-objectification, career aspirations, self-esteem, career decision-making self-efficacy, Chinese women

## Abstract

This study explored the relationship between self-objectification and career aspirations among young women from the perspective of objectification theory. A sample of 439 Chinese undergraduate women completed questionnaires on self-objectification, self-esteem, career decision-making self-efficacy, and career aspirations. The results revealed that self-objectification was negatively correlated with self-esteem, career decision-making self-efficacy, and career aspirations. Self-esteem and career decision-making self-efficacy, both independently and serially, mediated the association between self-objectification and career aspirations. These results provide a better understanding of the negative consequences of self-objectification for career aspirations.

## Introduction

Career aspirations reflect the extent to which an individual aspires to leadership positions and further education in their career (O’Brien et al., 2000). Research has shown that the levels for women’s career aspirations are significantly lower than those for men (e.g., Fritz and van Knippenberg, 2017), and low career aspirations hinder women’s career development and the realization of gender equality in the workplace (Crompton and Lyonette, 2011; Huang et al., 2021). The external environments, including family and work environments, are factors that influence women’s career aspirations (Pas et al., 2008; Fritz and van Knippenberg, 2017; Al-Bahrani et al., 2021). Recent studies have found that a social culture that values appearance also affects women’s career aspiration (Wang et al., 2020; Huang et al., 2021). These studies have also provided a new perspective for research in the field of women’s career development, suggesting that the sociocultural focus and gaze on women’s bodies may subtly affect women’s judgment of self-worth and thus affect their career development. Research has found that in this cultural atmosphere of emphasizing appearance, women tend to internalize ideal beauty or values emphasizing appearance, and then they pay more attention to their appearance, measure their value by appearance, and form self-objectification (Fredrickson and Roberts, 1997). Self-objectification causes women to define and evaluate themselves more based on observable physical attributes (e.g., weight) and ignore unobservable traits (e.g., health), which may also affect women’s career development (Daniels et al., 2020). Therefore, drawing on this perspective, this study explored the mechanisms through which self-objectification influences the career aspirations of female college students.

### Self-objectification and career aspiration

Fredrickson and Roberts (1997) argued that women are objectified when their bodies are perceived to represent them or when their value is equated with their bodies. According to objectification theory, by living in an environment that values appearances, women internalize an observer’s view of themselves, treating their bodies as objects based on appearance evaluation; this is called self-objectification (Fredrickson and Roberts, 1997). Studies have shown that a culture of objectifying women that focuses excessively on appearance constructs a negative and unsupportive environment for women’s development (Calogero, 2013; Huang et al., 2021) and limits their expectations and career choices (Daniels et al., 2020). For example, girls who played appearance-focused games (e.g., sexy Barbie dolls) reported fewer career choices and more identification with female careers than girls who played appearance-less games (e.g., Mrs. Potato Head) (Sherman and Zurbriggen, 2014; Slater et al., 2017). Similarly, in a sample of Chinese female college students, Huang et al. (2021) found that the more those young women agreed about the importance of physical attractiveness, the lower their reported levels of career aspirations. Self-objectification, as a result of the internalization of culturally driven objectification, may also have a negative impact on women’s career aspirations. Studies have found that self-objectifying women are used to constantly monitoring their bodies and paying more attention to their appearance; thus, they are more inclined to focus their time and energy on improving their physical attractiveness, while ignoring their own personality, strengths, and interests (Fredrickson and Roberts, 1997; Cao et al., 2021), which in turn may lower their career aspirations (Huang et al., 2021). An experimental study found that women who were objectified by male interactive partners experienced greater states of self-objectification and diminished career aspirations (Garcia et al., 2016). We therefore proposed that self-objectification would be negatively correlated with career aspirations (Hypothesis 1).

### Self-esteem as a potential mediator

Self-esteem is based on self-evaluation, and is a positive or negative attitude towards specific things related to the self (Rosenberg, 1965). A culture of objectification tells women that their body or their appearance is their most important attribute, and that those who are closer to realizing the cultural ideal of beauty are more valuable (Fredrickson and Roberts, 1997). Women engaged in self-objectification are more likely to evaluate themselves negatively through comparison with social standards or ideals; therefore, they tend to experience low self-esteem (Choma et al., 2010; Guo and Wu, 2021). Studies have shown that self-esteem can positively predict career aspirations (Castro and Armitage-Chan, 2016; Khampirat, 2020). Therefore, we proposed that self-objectification may be related to lower career aspirations via decreased self-esteem (Hypothesis 2).

### Career decision-making self-efficacy as a potential mediator

Career decision-making self-efficacy refers to an individual’s belief in their ability to achieve career results (Taylor and Betz, 1983). Studies have shown that low self-efficacy expectations are an important factor limiting women’s career choices (Betz and Hackett, 1981). Generally, women tend to lack a strong expectation of personal efficacy in many career-related behaviors, thus failing to fully realize their abilities and talents in career pursuits (Betz and Hackett, 1981; Beyer, 2014). For example, traditional views on gender roles and early learning experience limit women’s expectations of success and reduce their sense of professional self-efficacy, thus narrowing the scope of female career exploration and making them avoid choosing non-traditional female career fields, especially those involving mathematics, science, mechanical operation, and similar fields (Betz and Hackett, 1981; Taveira, 1997). From a cognitive perspective, some scholars believe that self-objectifying girls focus on body monitoring and thus consume their cognitive resources, thereby limiting their cognitive ability for other activities (Fredrickson et al., 1998), reducing their overall sense of self-efficacy. Studies have found that self-objectification is significantly negatively correlated with women’s self-efficacy (Gapinski et al., 2003; Adams et al., 2017). An experiment has shown that self-objectification induced by trying on swimsuits reduced women’s intrinsic motivation and self-efficacy (Gapinski et al., 2003). Career decision self-efficacy is a kind of self-efficacy, and we speculate that self-objectification may be related to low career decision-making self-efficacy. Research has shown that career decision self-efficacy serves as a positive predictor of career aspirations (Gregor et al., 2020; Al-Bahrani et al., 2021). Accordingly, we proposed that self-objectification may be related to lower career aspirations via decreased career decision-making self-efficacy (Hypothesis 3).

### Self-esteem and career decision-making self-efficacy

The above theoretical analysis shows that self-esteem and career decision-making self-efficacy may be the effective mediating variables between self-objectification and career aspirations. There is also a close relationship between self-esteem and career decision-making self-efficacy. A meta-analysis of career decision-making self-efficacy of Chinese college students shows that self-esteem has the highest correlation with career decision-making self-efficacy (*r* = 0.63), and high self-esteem helps to improve individual career decision-making self-efficacy (Li et al., 2016). Studies have shown that self-esteem can positively predict college students’ career decision-making self-efficacy (Thompson et al., 2019; Xu et al., 2021). Individuals with high self-esteem are more inclined to consider their own future and believe that they can solve work-related problems, which increases their confidence in career decisions (Xu et al., 2021); thus, they are more likely to have higher career aspirations (Al-Bahrani et al., 2021). Accordingly, we proposed that self-objectification may be related to lower career aspirations via decreased self-esteem and career decision-making self-efficacy (Hypothesis 4).

## Methods

### Participants and procedures

Our sample consisted of 439 women recruited from two universities in Henan, China. Participants ranged in age from 16 to 21 years (*M* = 18.26, *SD* = 0.83), and 97.49% were of Han ethnicity.

The study and the data collection procedure received approval from the ethics committees of the authors’ affiliate institutions. Participants were recruited from various elective psychology courses and received extra credit for their participation. Participants completed an online survey in Mandarin via Wenjuanxing (a Chinese survey website). Informed consent forms were submitted online before filling out the questionnaire.

### Measures

#### Self-objectification via body surveillance

Self-objectification was measured using the body surveillance subscale of the objectified body consciousness scale (OBCS, McKinley and Hyde, 1996). The OBCS includes three factors: body surveillance (habitually monitoring of one’s own body and viewing it as an outsider), body shame (feeling shame when the body does not conform to cultural beauty standards), and control beliefs (the belief that individuals can control how they look given enough effort) (McKinley and Hyde, 1996). Within the framework of objectification theory, the phenomenon of self-objectification enhances the perception that appearance is important to women, leading women to habitually monitor their own bodies from an outsider’s perspective (Fredrickson and Roberts, 1997). It can be seen that self-objectification is a similar construct to body surveillance proposed by McKinley and Hyde (1996). Therefore, the body surveillance subscale of the objectified body consciousness scale is widely used to measure women’s self-objectification and has been proved to have good reliability and validity (Moradi and Huang, 2008; Daniels et al., 2020). This subscale contains eight items that assess the frequency with which participants monitor their physical appearance. Participants responded to each item on a seven-point Likert scale ranging from 1 (*strongly disagree*) to 7 (*strongly agree*). The scale has satisfactory reliability and validity in Chinese female college students (Jackson and Chen, 2015). In this study, *α* =0.79.

#### Self-esteem

Consistent with previous studies (Choma et al., 2010; Guo and Wu, 2021), self-esteem was measured using the 10-item Rosenberg Self-esteem Scale (Rosenberg, 1965). Participants responded to each item on a four-point Likert scale ranging from 1 (*strongly disagree*) to 4 (*strongly agree*). The Chinese version showed satisfactory reliability and validity in Chinese samples (Wang et al., 1998). In this study, *α* = 0.87.

#### Career decision-making self-efficacy

Career decision-making self-efficacy was measured using the career decision-making self-efficacy scale-short form (CDSES-SF) (Betz et al., 1996). The scale comprises 25 items, with 5 items in each of 5 dimensions: accurate self-appraisal, gathering occupational information, goal selection, making plans for the future, and problem solving. Participants responded to each item on a five-point Likert scale ranging from 1 (*no confidence at all*) to 5 (*complete confidence*). The Chinese version showed satisfactory reliability and validity in Chinese samples (Kuang et al., 2011). As these five sub-scales were highly correlated with each other (*r* = 0.68 to 0.79, *p* < 0.001), in light of previous studies (Kuang et al., 2011), we combined all the items into a global indicator of career decision-making self-efficacy (*α* =0.94).

#### Career aspirations

Career aspirations were measured using the 8-item career aspiration scale (Gray and O’Brien, 2007). Participants responded to each item on a five-point Likert scale ranging from 0 (*strongly disagree*) to 4 (*strongly agree*). The Chinese version showed satisfactory reliability and validity in Chinese samples (Huang et al., 2021). In this study, *α* = 0.73.

### Data analysis

To test Hypothesis 1, we used SPSS version 20 to analyze the internal consistency, descriptive statistics, and correlations between the variables. To test Hypotheses 2–4, we first used structural equation modeling to test the hypothesized model. Next, we used PROCESS Model 6 (Hayes, 2013) to further test the significance of each mediation effect proposed in Hypotheses 2, 3, and 4. Bootstrapping analyses were used with 5,000 bootstrap samples to compute 95% bias-corrected.

## Results

The post-hoc power analysis using G*Power revealed that the large sample (*N* = 439, effect size = 0.3) provided sufficient power (around 100%) to detect key findings, using an alpha level of 0.05.

Table 1 shows the means, standard deviations, and correlations for all variables. Considering Hypothesis 1, the results show that self-objectification was negatively correlated with self-esteem, career decision-making self-efficacy, and career aspiration.

**Table 1:** Descriptive statistics and correlation among study variables (*N* = 439).

Variables	*M* (SD)	Min	Max	Range	1	2	3	4
1.Self-objectification	4.00 (0.91)	1.00	6.38	1–7	–	–	–	–
2. Self-esteem	2.96 (0.44)	1.60	4.00	1–4	−0.27^**^	–	–	–
3. Career decision-making self-efficacy	3.13 (0.54)	1.56	5.00	1–5	−0.30^**^	0.59^**^	–	–
4. Career aspirations	2.53 (0.53)	0.75	4.00	0–4	−0.17*	0.30^**^	0.36^**^	–

^*^p < 0.05, ^**^p < 0.01, ^***^p < 0.0001.

### Testing for mediation effect

To further test the validity of our model, some alternative mediation models were also tested: complete mediation model (M1), parallel multiple mediator model (M2), and chain mediation model (M3). The complete mediation model (M1) refers to self-objectification that affects career aspirations through self-esteem and career decision-making self-efficacy; parallel multiple mediator model (M2) is based on (M1), adding a direct impact path of self-objectification on career aspirations; the chain mediation model (M3) is a direct influence path of adding self-esteem to career decision-making self-efficacy on the basis of (M2). Results indicated a poor fit of the data to the three models (M1: *χ*^2^/*df* = 81.93, CFI = 0.46, GFI = 0.87, TLI = −0.62, SRMR = 0.17, RMSEA = 0.43; M2: *χ*^2^*/df* = 162.57, CFI = 0.46, GFI = 0.87, TLI = −2.23, SRMR = 0.17, RMSEA =0.61; M3: *χ*^2^/*df* = 0.00, CFI = 1.00, GFI = 1.00, TLI = 0.00, SRMR = 0.00, RMSEA = 0.34), and the path from self- objectification to career aspirations is not significant (M1: *p* = 0.26; M2: *p* = 0.27). We deleted the non-significant paths and re-analyzed the model 3 (the chain mediation model); the fit statistics indicated a good fit to the data (*χ*^2^/*df* = 1.29, CFI = 0.99, TLI = 0.99, SRMR = 0.02, RMSEA = 0.03). This finding corroborates the validity of our hypothesis.

The regression results of the sample confirmed that: (1) self-objectification negatively predicted self-esteem (*β* = −0.27, *p* < 0.001); (2) self-esteem had a positively prediction on career decision-making self-efficacy (*β* = 0.55, *p* < 0.001); and (3) career decision-making self-efficacy positively predict career aspirations (*β* = 0.27, *p* < 0.001). Then, self-esteem and career decision-making self-efficacy were added in, self-objectification did not predict career aspirations (*β* = −0.005, *p* = −1.19).

**Figure 1:**
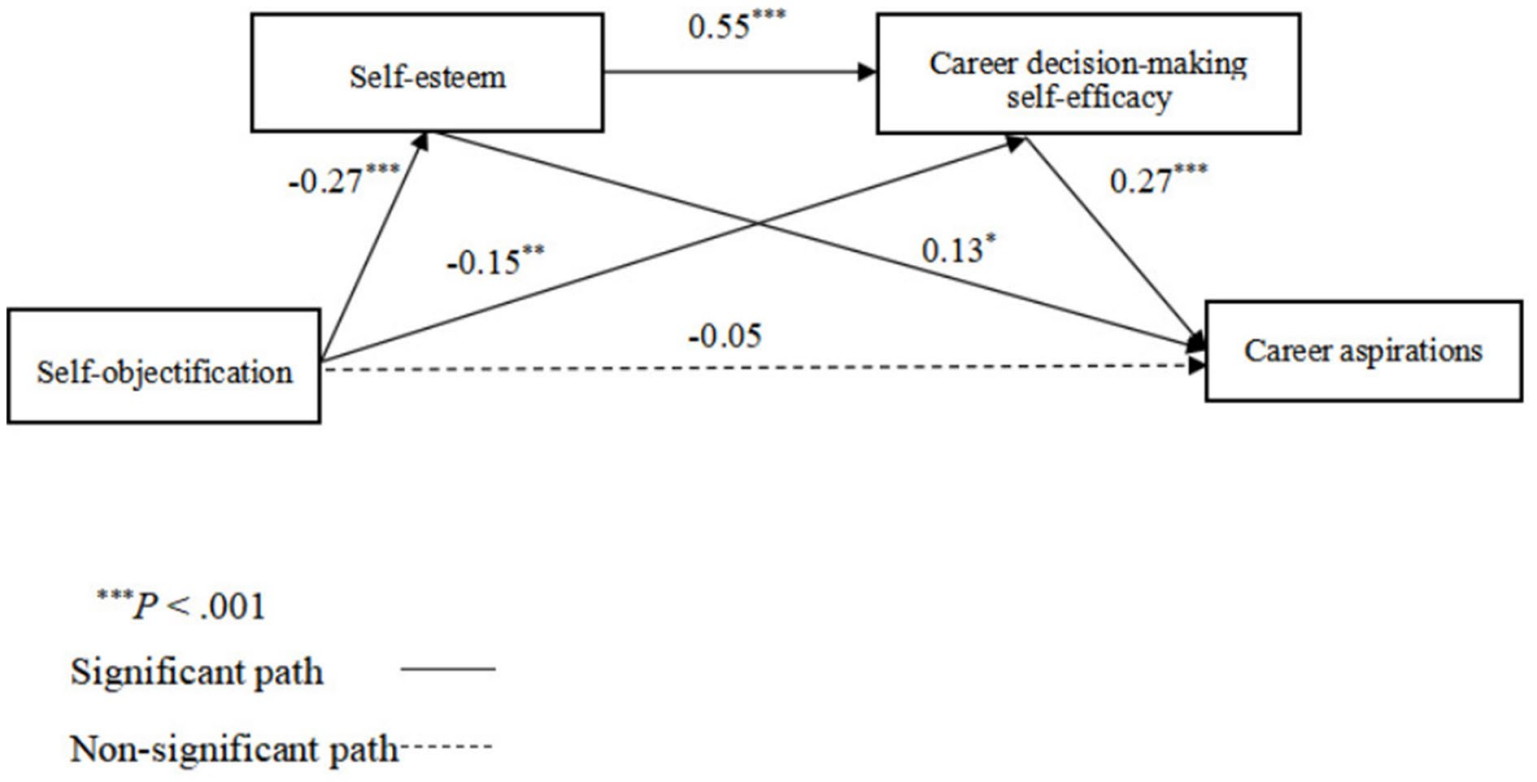
Hypothesized and final model with standardized path coefficients.

The mediation analysis showed that self-esteem mediated the association between self-objectification and career aspirations [indirect effect =0.020, *SE* = 0.010, 95% CI (−0.041, −0.001)] (supporting Hypothesis 2), as did career decision-making self-efficacy [indirect effect =0.023, *SE* = 0.009, 95% CI (−0.043, −0.009)] (supporting Hypothesis 3). The results also supported the serial mediating roles of self-esteem and career decision-making self-efficacy in the association between self-objectification and career aspirations [indirect effect = −0.024, SE =0.007, 95%CI (−0.038, −0.012)], supporting Hypothesis 4 (see Figure 1).

## Discussion

Although previous studies have found that a culture focused on appearance has a negative impact on young women’s career aspirations (Wang et al., 2020; Huang et al., 2021), few studies have directly explored the relationship between self-objectification and career aspirations directly, and the mechanism connecting the two remains unclear. This study examined this relationship and found that self-objectification is significantly negatively correlated with career aspirations. This result is consistent with previous studies and with our hypothesis, indicating that women with higher levels of self-objectification are more likely to have lower career aspirations (Garcia et al., 2016). Self-objectifying women pay more attention to their appearance and ignore their inner qualities (Fredrickson and Roberts, 1997; Cao et al., 2021); they may be less likely to explore their interests, values, and careers scope, which can inhibit their career aspirations. A culture of objectification also overstates the value of women’s appearance and body (Huang et al., 2021); for example, in the media, a perfect appearance is often associated with personal value and success (Luo, 2012). A women’s beautiful body is regarded as a form of capital or competitive advantage to obtain more social resources and opportunities to change one’s life (Calogero, 2013; Wang et al., 2020). In such a culture, women tend to experience greater employment pressure and perceive fewer opportunities, reducing their career aspirations (Huang et al., 2021). Women who experience self-objectification are more likely to be affected by this culture (Calogero, 2013), as the unattainably perfect body may make them feel that they lack value or competitiveness, which may also curb their career aspirations.

As predicted, self-esteem mediates the relationship between self-objectification and career aspirations. Self-objectifying women tend to constantly monitor their bodies and are more likely to be compared to society’s ideal standards of beauty. The gap between the two leads to negative self-evaluation and thus such women tend to experience low self-esteem (Choma et al., 2010; Guo and Wu, 2021). Low self-esteem in turn may make women take a less positive view of their learning and skill development, which can prevent them from pursuing leadership roles, thus limiting their career aspirations (Castro and Armitage-Chan, 2016). Although the self-esteem scores of women in this study were not as low (*M* = 2.96, SD = 0.44), they were similar to those found in previous studies. For example, in the study of Tang et al. (2019), the average self-esteem score of Chinese female college students was 2.95 (SD = 0.42), and in the study of Choma et al (2010), the average self-esteem score of Canadian female students was 3.15 (SD = 0.48). The reason why the level of self-esteem is not low enough may be because the personal self-value assessment is complex and may be affected by many factors (Fang et al., 2016). In this study, we pay more attention to the relationship between various variables and self-esteem, especially the negative impact of self-objectification on female self-esteem. Our results demonstrated a negative correlation between self-objectification and self-esteem, while low self-esteem further affects women’s career aspirations.

Career decision-making self-efficacy also mediated the relationship between self-objectification and career aspirations. This result extends the negative impact of self-objectification on women’s self-efficacy to the career field (Adams et al., 2017). Self-objectifying women’s habitual monitoring of their bodies occupies psychological resources, which makes it difficult for them to focus on other things (Fredrickson et al., 1998); this may reduce their self-efficacy. Self-objectification can also lead women to ignore the development of or dramatically underestimate their abilities (Loughnan et al., 2017; Cao et al., 2021). This neglect and underestimation of their abilities may reduce women’s confidence in completing certain activities or behaviors, which in turn may lead to low career decision-making self-efficacy. Individuals with low career decision-making self-efficacy tend to show lower career aspirations (Al-Bahrani et al., 2021). Women with self-objectification are thus more likely to show low self-efficacy in career decision-making, which limits their career aspirations.

Finally, our findings add value to the existing literature by suggesting the serial mediation of self-esteem and career decision-making self-efficacy in the relationship between self-objectification and career aspiration. This result provides possible pathways to explain the relationship between self-objectification and career aspiration. Based on objectification theory, self-objectifying women regard their physical appearance as the criterion for judging their own value (Fredrickson and Roberts, 1997). From this perspective, self-objectifying women are more likely to exhibit lower self-esteem and self-worth (Adams et al., 2017; Guo and Wu, 2021), as they are unable to fully and accurately evaluate themselves. They may then have more difficulties and exhibit lack of confidence in career decision-making, which ultimately undermines their career aspirations. This finding helps to deepen our understanding of the negative effects of self-objectification on women’s career development.

### Implications for practice

First, our results enrich the existing research on objectification theory, indicating that self-objectification has a wide range effects and may affect many aspects of women’s lives. Second, this study broadens the horizons for research on women’s careers and provides evidence that objectifying women is a form of gender bias that creates a negative environment for women’s development (Calogero, 2013; Calogero and Tylka, 2014; Huang et al., 2021). This cultural pressure that encourages women to pursue beauty rather than achievement promotes women viewing themselves from the perspective of self-objectification. This limits women’s roles and further consolidates their vulnerable position in the gender hierarchy (Calogero, 2013). Society and the media should thus reduce the objectification of and present more diverse female images to create a healthy environment for female development. Finally, this study provides further evidence that self-esteem and career decision-making self-efficacy mediate how self-objectification affects women’s career ambition, which has implications for the career planning education. Female college students could be encouraged, for example, to focus on the development of their inner qualities and abilities to improve their self-esteem and sense of self-worth, thus promoting better career development.

### Limitations

The limitations of the current study are as follows. First, this was a cross-sectional study, meaning that we cannot reveal the causal relationship between the variables; experimental and longitudinal studies are therefore needed. Second, our results are based on responses from Chinese female college students, which mean that the results may not be generalizable to women from other countries. Future studies should thus expand the range of their samples to improve the external validity of the results. Finally, this study only discussed the mediating mechanism between self-objectification and career aspirations, without involving individual differences. A study has found a significant positive relationship between body surveillance and career aspirations, which is inconsistent with our findings (Wang et al., 2020). The “beauty as currency” hypothesis holds that beauty can be used as the social currency of women (Calogero et al., 2017). Thus, one possible explanation is that for highly attractive self-objectifying women, beauty may be seen as a useful form of social capital or competitive advantage, and may lead to higher career aspirations. Future research could further explore the whether beauty as currency belief and evaluation of self-attractiveness moderate the effects of self-objectification on women’s career aspirations.

## Conclusion

This study explored the association between self-objectification and career aspirations, as well as the underlying mechanisms in this relationship, among young Chinese women. The data obtained supported our hypotheses that Chinese female college students with higher self-objectification would be more likely to have lower career aspirations and that this relationship is mediated by self-esteem and career decision-making self-efficacy. These results help us to better understand the mechanisms through which self-objectification affects women’s career aspirations.

## Data availability statement

The raw data supporting the conclusions of this article will be made available by the authors, without undue reservation.

## Ethics statement

The studies involving human participants were reviewed and approved by the Ethics Committee of Henan University of Economics and Law. Written informed consent to participate in this study was provided by the participants’ legal guardian/next of kin.

## Author contributions

QS carried out the experimental work and the data collection, interpretation, and wrote the manuscript.

## Conflict of interest

The author declares that the research was conducted in the absence of any commercial or financial relationships that could be construed as a potential conflict of interest.

## Publisher’s note

All claims expressed in this article are solely those of the authors and do not necessarily represent those of their affiliated organizations, or those of the publisher, the editors and the reviewers. Any product that may be evaluated in this article, or claim that may be made by its manufacturer, is not guaranteed or endorsed by the publisher.
